# Optimization of management plan with a machine learning model for ovarian torsion cases: operative vs. conservative

**DOI:** 10.3389/fmed.2026.1742184

**Published:** 2026-06-25

**Authors:** Alia Alethawy, Omaima Al-Baghdadi, Yauhen Statsenko, Moamar Al-Jefout

**Affiliations:** 1 Department of Obstetrics and Gynecology, Tawam Hospital, Al Ain, United Arab Emirates; 2 Department of Radiology, College of Medicine and Health Sciences, United Arab Emirates University, Al Ain, United Arab Emirates; 3 Department of Obstetrics and Gynecology, College of Medicine and Health Sciences, United Arab Emirates University, Al Ain, United Arab Emirates

**Keywords:** ovarian torsion, machine learning, prognostic model, conservative management, gynecologic emergencies, fertility preservation

## Abstract

**Background:**

Ovarian torsion is a gynecologic emergency requiring timely management to preserve ovarian function and future fertility. Clinical presentation is heterogeneous and frequently non-specific, making the decision between operative and conservative management challenging, particularly in reproductive-aged patients.

**Objective:**

Develop and evaluate a machine learning–based prognostic model to support individualized management decisions in ovarian torsion.

**Methods:**

We conducted a retrospective cohort study of 219 patients diagnosed with ovarian torsion. Demographic characteristics, reproductive and surgical history, comorbidities, presenting symptoms, laboratory findings, and imaging features were analyzed. Multiple supervised machine learning models were trained and compared, including Logistic Regression, Decision Tree, Random Forest, Gradient Boosting, and Neural Network classifiers. Class imbalance was addressed using class weighting and oversampling techniques (ROSE, SMOTE, SMOTE-ENN). Model performance was evaluated using area under the receiver operating characteristic curve (AUC), sensitivity, and specificity. Principal component analysis (PCA) and unsupervised clustering were performed to identify latent clinical domains and phenotypic subgroups.

**Results:**

The class-weighted Decision Tree demonstrated the most balanced and clinically interpretable performance (AUC = 0.76; sensitivity = 0.75; specificity = 0.73). Key predictors included doppler findings, BMI, blood group (ABO), ethnicity, major and associated clinical symptoms, the presence of pelvic mass, menopause, history of OCP, infertility and previous surgery. PCA identified eight clinical domains, and clustering revealed two phenotypically distinct patient profiles, underscoring the heterogeneity of ovarian torsion presentations.

**Conclusion:**

Machine learning models can support individualized risk stratification in ovarian torsion. A transparent, non-overfitted Decision Tree model offers a clinically interpretable framework that may assist fertility-preserving management decisions. Prospective multicenter validation is required before clinical implementation.

## Introduction

Ovarian torsion (OT) is a gynecological emergency that requires prompt diagnosis and management to preserve ovarian function and prevent serious complications. OT, when it is associated with tubal torsion, is termed an adnexal torsion. The annual prevalence is estimated to be around 9.9/100,000 women of reproductive age (1). The pathophysiology of OT involves torsion of the ovary on its pedicle, leading to compromised venous return, stromal swelling, internal bleeding, and ischemia, which may lead to necrosis. Timely and accurate decisions regarding surgical intervention vs. conservative management are crucial for preserving the reproductive function of women with this pathology. Classical diagnostic criteria focus heavily on clinical signs such as abdominal pain, nausea, and vomiting (2), which are normal clinical presentations, and imaging (Doppler scan usually shows reduced ovarian blood supply), which may not fully capture the complexity of torsion cases.

Ovarian torsion is usually managed surgically, normally in the form of diagnostic laparoscopy, which is the gold standard for the diagnosis of OT; furthermore, it enables treatment, either by oophorectomy or detorsion, although recent publications showed a modest decrease in the proportion of oophorectomies for ovarian torsion (3). However, there is still a risk of serious complications from laparoscopy in approximately 2 per 1,000 cases (4), which can include organ trauma or major vessel injury, each carrying significant morbidity and mortality (5).

There is some confusion in the literature regarding the factors responsible for the development of ovarian torsion, and what the odds are of a particular clinical feature in determining the likelihood of developing ovarian torsion. Moreover, clinical decision-making is often challenged by non-specific symptoms and overlapping features with other acute abdominal conditions. Hence, it is vital to develop a prognostic Model Management Plan (MMP) determining either operative or conservative (non-surgical) management for cases with ovarian torsion. Machine Learning (ML) methods offer the potential to enhance prognostic accuracy by analyzing large, multidimensional clinical datasets, and uncovering non-linear patterns that may not be evident to clinicians. The application of ML to ovarian torsion may facilitate the development of predictive models that inform real-time decision-making, guiding whether operative or conservative management is more appropriate for individual patients. This study aims to develop and evaluate ML-based models that predict management outcomes in ovarian torsion using a diverse clinical dataset (6, 7).

Our study aims to achieve four objectives: **Objective 1** is to estimate the differences in the demographics, clinical histories, and clinical data between the groups with conservative and operative management. **Objective 2:** to identify the most informative clinical, demographic, and historical features influencing the management decision. **Objective 3:** to assess relationships among studied variables and find their associations reflecting the risk factors of ovarian torsion, clinical symptoms, and disease management. **Objective 4**: to build a model prognosticating a suitable disease management plan (operative vs. conservative).

### Data collection and preprocessing

Data were extracted from electronic medical records, and they included demographic characteristics, reproductive and surgical history, comorbidities, presenting symptoms, laboratory values, and imaging findings. Missingness was assessed for each variable prior to analysis. Variables with substantial missingness were not used for supervised model training. For retained predictors, missing continuous values were treated with median imputation, and missing categorical values were treated with mode imputation. To minimize data leakage, imputation was performed within each training fold during cross-validation, with imputation parameters estimated on the training data and applied to the corresponding validation fold. A complete-case analysis was additionally performed as a sensitivity check to confirm that imputation did not materially alter model performance.

### Machine learning modeling

We developed supervised machine learning models to classify ovarian torsion cases according to management strategy (operative vs. conservative). Predictor variables included demographic characteristics, reproductive and surgical history, comorbidities, presenting symptoms, laboratory values, and imaging findings, consistent with prior observational studies in ovarian torsion (16).

Given the marked class imbalance, several imbalance-handling strategies were explored, including class weighting and synthetic oversampling techniques (ROSE, SMOTE, and SMOTE-ENN), which are commonly applied in imbalanced biomedical datasets (15). Model performance was evaluated using stratified k-fold cross-validation.

We trained Logistic Regression, Decision Tree, Random Forest, Gradient Boosting, and Neural Network classifiers. Performance metrics included AUC, sensitivity, specificity, and accuracy. Model selection emphasized robustness and interpretability, particularly considering known overfitting risks in small, high-dimensional clinical datasets (18).

### Principal component analysis and clustering

Principal component analysis (PCA) was performed to identify latent clinical domains underlying ovarian torsion presentation. Variables were standardized prior to PCA, and components with eigenvalues greater than one were retained. Varimax rotation was applied to enhance interpretability, consistent with established exploratory analytic approaches in clinical phenotyping studies (17).

Unsupervised k-means clustering was subsequently applied to identify phenotypic patient groups, with the optimal number of clusters determined using silhouette and gap statistic methods. Clustering was used to explore clinical heterogeneity rather than to define management pathways.

## Objectives

### Objective 1: estimate the differences in the demographics, clinical histories, and clinical data between the groups with conservative and operative management

#### Methodology

In the first objective, we assessed the differences in between the demographics, clinical histories, and clinical data between the groups with conservative and operative management. To make the comparison, we used the Mann-Whitney U test due to the non-Gaussian distribution of variables.

A total of 219 women were included in the study, of whom 36 (16.4%) were managed conservatively and 177 (80.8%) underwent operative intervention. A subgroup analysis was performed on 179 patients with complete reproductive data.

#### Results

Table 1 compares demographic, clinical, surgical, and outcome variables between two treatment strategies for ovarian torsion: Conservative management (*n* = 36) and Operative management (*n* = 183). It also stratifies operative cases into laparotomy (*n* = 28) and laparoscopy (*n* = 155). Continuous variables are presented as mean ± SD, categorical variables as % (n). *P-values* reflect comparisons between groups.

**Table 1:** Comparison of demographic, clinical, and surgical characteristics between conservative and operative treatment groups in ovarian torsion.

Variable	Unit	Total sample (*n_1_* = 219), M_1_ ± SD_1_ (1)	Conservative treatment (*n_2_* = 36), M_2_ ± SD_2_ (2)	Operative treatment (*n_3_* = 183), M_3_ ± SD_3_ (3)	*p*_2 − 3_	Laparotomy (*n_4_*= 28), M_4_ ± SD_4_ (4)	Laparoscopy (*n_5_* = 155), M_5_ ± SD_5_ (5)	*p*_4 − 5_
Age	year	28.28 ± 12.37	28.25 ± 10.80	28.29 ± 12.69	< 0.001	38.43 ± 17.50	26.46 ± 10.70	< 0.001
BMI	kg/m^2^	26.80 ± 6.28	25.68 ± 5.45	27.02 ± 6.43	0.058	29.15 ± 6.82	26.64 ± 6.30	0.058
Menopause	% (*n*)	10.5 (23)	5.56 (2)	11.48 (21)	0.040	32.14 (9)	7.74 (12)	< 0.001
Gravidity	*n*	1.75 ± 2.62	2.00 ± 2.59	3.39 ± 2.63	0.004	3.14 ± 4.13	1.45 ± 2.17	0.002
Parity	*n*	1.24 ± 2.00	1.44 ± 2.30	1.20 ± 1.94	0.003	2.50 ± 3.27	0.96 ± 1.49	< 0.001
Miscarriage	*n*	0.32 ± 1.07	0.31 ± 0.75	0.33 ± 1.12	0.874	0.46 ± 1.35	0.30 ± 1.08	0.786
Surgical history								
Previous surgeries	*n*	0.35 ± 0.80	0.50 ± 1.18	0.32 ± 0.71	0.845	0.32 ± 0.68	0.32 ± 0.71	0.788
Previous cesarean sections	*n*	0.20 ± 0.67	0.36 ± 1.15	0.17 ± 0.53	0.830	0.18 ± 0.48	0.17 ± 0.54	0.703
Past medical and social history								
Smoking	% (*n*)	0.91 (2)	0.0 (0)	1.09 (2)	0.915	0.00 (0)	1.29 (2)	0.554
Pelvic surgery	% (*n*)	24.20 (53)	25.00 (9)	24.04 (44)	0.654	28.57 (8)	23.23 (36)	0.545
Salpingectomy and/or oophorectomy	% (*n*)	1.37 (3)	0.00 (0)	1.64 (3)	0.872	0.00 (0)	1.94 (3)	0.465
Appendectomy	% (*n*)	5.48 (12)	5.56 (2)	5.46 (10)	0.869	7.14 (2)	5.16 (8)	0.676
Cystectomy	% (*n*)	2.74 (6)	5.56 (2)	2.19 (4)	0.830	0.00 (0)	2.58 (4)	0.396
Ovarian and/or tubal detorsion	% (*n*)	2.74 (6)	2.78 (1)	2.73 (5)	0.935	3.57 (1)	2.58 (4)	0.773
Laparoscopic tubal ligation	% (*n*)	0.91 (2)	0.00 (0)	1.09 (2)	0.915	0.00 (0)	1.29 (2)	0.554
Other surgeries	% (*n*)	1.37 (3)	0.00 (0)	1.64 (3)	0.849	3.57 (1)	1.29 (2)	0.388
Cesarean section	% (*n*)	12.33 (27)	13.89 (5)	12.02 (22)	0.824	14.29 (4)	11.61 (18)	0.692
Endometriosis	% (*n*)	0.46 (1)	0.00 (0)	0.55 (1)	0.958	0.00 (0)	0.65 (1)	0.682
OCP	% (*n*)	10.96 (24)	25.00 (9)	8.20 (15)	0.918	7.14 (2)	8.39 (13)	0.829
PID	% (*n*)	1.37 (3)	0.00 (0)	1.64 (3)	0.872	0.00 (0)	1.94 (3)	0.465
Ovarian torsion	% (*n*)	5.94 (13)	5.56 (2)	6.01 (11)	0.912	7.14 (2)	5.81 (9)	0.788
Comorbidities								
Total number	*n*	1.00 ± 0.93	1.06 ± 0.86	0.99 ± 0.94	0.314	1.11 ± 0.80	0.97 ± 0.97	0.288
Normal pregnancy	% (*n*)	19.18 (42)	19.44 (7)	19.33 (35)	0.901	17.86 (5)	19.35 (30)	0.856
Sleeve gastrectomy	% (*n*)	1.83 (4)	0.00 (0)	2.19 (4)	0.830	0.00 (0)	2.58 (4)	0.396
PCOS (includes all cases of “history of infertility”)	% (*n*)	12.33 (27)	19.44 (7)	10.93 (20)	0.985	10.71 (3)	10.97 (17)	0.971
IVF	% (*n*)	3.65 (8)	8.33 (3)	2.73 (5)	0.788	0.00 (0)	3.23 (5)	0.340
IVF with OHSS	% (*n*)	2.28 (5)	2.78 (1)	2.19 (4)	0.830	0.00 (0)	2.58 (4)	0.396
Pelvic mass (ovarian cyst and other masses)	% (*n*)	30.59 (67)	16.67 (6)	33.33 (61)	0.098	50.00 (14)	30.32 (47)	0.043
IUCD	% (*n*)	1.83 (4)	2.78 (1)	1.64 (3)	0.872	0.00 (0)	1.94 (3)	0.465
Endometriosis	% (*n*)	2.28 (5)	5.56 (2)	1.64 (3)	0.849	3.57 (1)	1.29 (2)	0.388
Obesity	% (*n*)	1.37 (3)	2.78 (1)	1.09 (2)	0.915	0.00 (0)	1.29 (2)	0.554
Metabolic syndrome	% (*n*)	0.46 (1)	0.00 (0)	0.55 (1)	0.765	3.57 (1)	0.00 (0)	0.019
History of pelvic surgery	% (*n*)	24.66 (54)	27.78 (10)	24.04 (44)	0.926	25.00 (7)	23.87 (37)	0.900
Major symptoms								
Total number	*n*	1.04 ± 0.19	1.00 ± 0.00	1.04 ± 0.20	0.530	1.11 ± 0.31	1.03 ± 0.18	0.076
Flank pain	% (*n*)	2.28 (5)	2.78 (1)	2.18 (4)	0.830	0.00 (0)	2.58 (4)	0.396
Abdominal distension	% (*n*)	3.65 (8)	0.00 (0)	4.37 (8)	0.530	10.71 (3)	3.23 (5)	0.076
Epigastric pain	% (*n*)	0.46 (1)	0.00 (0)	0.55 (1)	0.958	0.00 (0)	0.65 (1)	0.682
Generalized abdominal pain	% (*n*)	1.83 (4)	2.78 (1)	1.64 (3)	0.872	0.00 (0)	1.94 (3)	0.465
Unilateral lower abdominal pain	% (*n*)	90.87 (199)	91.67 (33)	90.71 (166)	0.571	96.43 (27)	89.68 (139)	0.260
Pelvic pain	% (*n*)	4.57 (10)	2.78 (1)	4.92 (9)	0.895	3.57 (1)	5.16 (8)	0.725
Associated symptoms								
Total number	*n*	0.66 ± 0.64	0.44 ± 0.56	0.69 ± 0.65	0.983	0.71 ± 0.71	0.70 ± 0.64	0.981
GI symptoms								
Nausea	% (*n*)	28.31 (62)	25.00 (9)	28.96 (53)	0.754	32.14 (9)	28.38 (44)	0.689
Vomiting	% (*n*)	39.73 (87)	30.56 (11)	41.53 (76)	0.101	25.00 (7)	44.51 (69)	0.055
Fainting	% (*n*)	0.46 (1)	0.00 (0)	0.55 (1)	0.958	0.00 (0)	0.64 (1)	0.682
Constipation	% (*n*)	3.20 (7)	2.78 (1)	3.28 (6)	0.703	7.14 (2)	2.58 (4)	0.216
Diarrhea	% (*n*)	1.83 (4)	2.78 (1)	1.64 (3)	0.872	0.00 (0)	1.94 (3)	0.465
Loss of appetite	% (*n*)	3.20 (7)	0.00 (0)	3.82 (7)	0.495	10.71 (3)	2.58 (4)	0.040
Flu-like symptoms								
Fever	% (*n*)	5.02 (11)	0.00 (0)	6.01 (11)	0.642	10.71 (3)	5.16 (8)	0.259
Shivering, shortness of breath, headache	% (*n*)	2.74 (6)	2.78 (1)	2.73 (5)	0.788	0.00 (0)	3.23 (5)	0.340
Vaginal symptoms								
Vaginal bleeding or discharge	% (*n*)	1.83 (4)	0.00 (0)	2.18 (4)	0.830	0.00 (0)	2.58 (4)	0.396
Dysuria	% (*n*)	2.28 (5)	0.00 (0)	2.73 (5)	0.935	3.57 (1)	2.58 (4)	0.773
Diagnostic modalities								
Total number	*n*	0.58 ± 0.66	0.47 ± 0.65	0.61 ± 0.66	0.012	1.00 ± 0.86	0.54 ± 0.59	0.005
Transvaginal ultrasound	% (*n*)	26.48 (58)	30.56 (11)	25.68 (47)	0.674	21.43 (6)	26.45 (41)	0.578
Abdominal ultrasound	% (*n*)	6.39 (14)	2.78 (1)	7.10 (13)	0.477	14.29 (4)	5.81 (9)	0.110
CT abdomen/pelvis	% (*n*)	19.63 (43)	8.33 (3)	21.86 (40)	0.037	42.86 (12)	18.06 (28)	0.004
MRI pelvis	% (*n*)	5.94 (13)	5.56 (2)	6.01 (11)	0.126	21.43 (6)	3.23 (5)	0.000
Disease management								
Onset-to-surgery time	h	1.80 ± 4.86	–	1.79 ± 4.86	–	3.16 ± 6.34	1.57 ± 4.55	0.083
Treatment outcomes								
AMH level after treatment	pmol/l	34.87 ± 27.07	17.18 ± 13.85	38.67 ± 28.01	1.000	–	38.67 ± 28.01	1.000
FSH level after treatment	IU/l	10.24 ± 19.05	4.46 ± 2.35	11.50 ± 20.82	0.091	14.65 ± 15.12	11.29 ± 21.23	0.091
LH level after treatment	IU/l	11.33 ± 12.62	4.80 ± 3.34	12.75 ± 13.45	0.346	14.88 ± 9.22	12.61 ± 13.72	0.346
Fecundity after treatment	% (*n*)	31.50 (69)	36.11 (13)	30.60 (56)	0.768	17.85 (5)	32.90 (51)	0.682
Torsion recurrence	% (*n*)	3.65 (8)	0.00 (0)	4.37 (8)	0.938	3.57 (1)	4.51 (7)	0.827

Demographic Characteristics: Age differed significantly between each two groups: conservative vs. operative treatment (*p* < 0.001) and laparotomy vs. laparoscopy (*p* < 0.001). In comparison, operative patients were slightly older on average, with laparotomy patients markedly older (38.4 ± 17.5 y) than laparoscopy patients (26.5 ± 10.7 y). BMI was somewhat higher in operative patients (27.0 ± 6.4) than in those patients with conservative management (25.7 ± 5.5), borderline significant (*p* = 0.058). We observed similar borderline difference between laparotomy and laparoscopy (*p* = 0.058). Menopause was more frequent in operative (11.5%) vs. conservative (5.6%) (*p* = 0.040), and much higher in laparotomy (32.1%) vs. laparoscopy (7.7%) (*p* < 0.001).

Obstetric History: Gravidity and parity were significantly higher in operative vs. conservative management (*p* = 0.004, *p* = 0.003), and higher in laparotomy vs. laparoscopy (*p* = 0.002, p < 0.001). Miscarriage rates showed no significant differences (*p* > 0.7).

Past Surgical/Medical History: The research revealed no significant differences for prior pelvic surgery, appendectomy, cystectomy, or cesarean section (all *p* > 0.3). History of endometriosis, OCP use, PID, or PCOS showed no group differences (all *p* > 0.7).

Comorbidities: Overall comorbidities count similarly across groups (*p* > 0.2). Metabolic syndrome was rare but significantly more frequent in laparotomy vs. laparoscopy (3.6% vs. 0%; *p* = 0.019).

Symptoms: Unilateral lower abdominal pain was the most frequent symptom (91%), with no significant group difference (*p* > 0.2). Vomiting was more common in operative than conservative management (41.5% vs. 30.6%; *p* = 0.101, NS) and trended lower in laparotomy vs. laparoscopy (25% vs. 44.5%; *p* = 0.055). Loss of appetite was significantly more frequent in the laparotomy group (10.7%) than in the laparoscopy group (2.6%) (*p* = 0.040).

Diagnostic Modalities: Conservative patients underwent fewer imaging investigations overall (mean number lower; *p* = 0.012). CT abdomen/pelvis and MRI pelvis were used more often in laparotomy vs. laparoscopy (*p* = 0.004 and *p* < 0.001, respectively).

Treatment Outcomes: Onset-to-surgery time was longer in laparotomy (3.16 h) vs. laparoscopy (1.57 h) (*p* = 0.083, NS). Hormonal markers [Anti-Müllerian Hormone (AMH); Follicle-Stimulating Hormone (FSH); Luteinizing Hormone (LH)] showed no significant differences between groups (all *p* > 0.09). Fecundity after treatment was similar between conservative (36.1%) and operative (30.6%) (*p* = 0.768). Torsion recurrence was rare (overall 3.7%) with no significant group differences.

In summary, highly significant differences (*p* < 0.001) were found for: age, menopause, gravidity, parity, and diagnostic modalities (CT, MRI). Moderately significant differences (*p* < 0.05) were found for: metabolic syndrome, loss of appetite, and – when comparing both main groups and surgical subgroups – for gravidity and parity. Borderline significant differences (*p* = 0.05–0.1) were found for: BMI, vomiting, onset-to-surgery time, and abdominal distension. Non-significant differences were found for: most comorbidities, surgical history, OCP/PCOS/infertility, recurrence rates, fecundity, and hormone levels (Table 1).

### Objective 2: identify the most informative clinical, demographic, and historical features influencing the management decision

#### Methodology

In the second objective, we looked for possible associations in demographics, clinical histories, clinical data, and patient management type. Since the data studied did not follow the Gaussian distribution, we tested how tightly they are linked by computing Spearman rank correlation criterion. The analysis sought to determine whether significant associations existed between the studied variables and the management approach—operative vs. conservative.

#### Results

Out of more than 60 tested variables, only three showed statistically significant associations (*p* < 0.05) with the treatment type:

1. History of Oral Contraceptive Pills (OCP) use (*r* = −0.199, *p* = 0.003)

Interpretation: There was a marked negative correlation between the history of OCP use and undergoing operative treatment. This suggests that patients with a history of OCP use were more likely to be managed conservatively. This could imply a protective or modifying role of OCPs in disease progression or symptom control.

2. Presence of Pelvic Mass (ovarian cyst or other masses) (*r* = 0.134, *p* = 0.048)

Interpretation: A weak positive correlation was observed, indicating that patients with pelvic masses were slightly more likely to undergo operative treatment. This aligns with clinical expectations, where structural pathology (e.g., cysts) often necessitates surgical intervention.

3. Number of Associated Symptoms (*r* = 0.148, *p* = 0.029)

Interpretation: A strong positive association indicates that having more concurrent symptoms may tip the balance toward surgical management, likely due to symptom severity or diagnostic ambiguity. All other parameters show no significant relationships.

### Objective 3: assess the relationships among the studied variables and find their associations reflecting the risk factors of ovarian torsion, clinical symptoms, and disease management

#### Methodology

Objective 3 aimed to explore complex interrelationships among demographic, clinical, and management-related variables in patients with ovarian torsion. A two-phase analytic approach was undertaken:

1. Principal component analysis (PCA).

2. Cluster analysis.

After standardization via mean normalization, PCA with Varimax rotation was performed to reduce dimensionality and identify latent structures in the data. Variables with eigenvalues ≥1.0 were retained. Loadings ≥0.75 were considered strong, 0.50–0.75 moderate, and < 0.50 weak (Table 2). This enabled the identification of key clinical syndromes and background features associated with ovarian torsion. Overall, the PCA structure supports the multidimensional nature of ovarian torsion presentations, capturing elements of age/reproductive history, acute symptomatology, systemic response, and surgical background. This factor structure was essential in informing the subsequent predictive modeling process.

**Table 2:** Principal component analysis: Eigenvalues and rotated factor loadings of significant variables.

Variable	Factor 1	Factor 2	Factor 3	Factor 4	Factor 5	Factor 6	Factor 7	Factor 8
Eigenvalue	6.759	3.737	3.189	2.841	2.714	2.446	2.412	2.262
% Total variance	10.729	5.932	5.062	4.509	4.308	3.883	3.828	3.591
Cumulative eigenvalue	6.759	10.497	13.685	16.526	19.240	21.687	24.098	26.360
Cumulative %	10.730	16.661	21.723	26.232	30.540	34.423	38.251	41.842
Factor loadings								
Age	−0.796	0.040	0.035	−0.100	−0.051	0.045	0.213	0.047
Ethnicity	0.130	−0.114	−0.251	−0.122	−0.065	0.110	0.059	−0.206
Marital status	0.544	−0.137	−0.135	−0.079	0.048	0.010	−0.261	−0.338
Blood ABO	−0.051	−0.037	0.033	0.039	−0.135	−0.180	−0.114	−0.055
Blood rhesus	0.097	−0.009	−0.025	−0.068	0.188	0.140	0.093	−0.271
BMI	−0.359	0.104	−0.218	−0.144	−0.115	0.300	0.129	0.313
Menopause	−0.686	0.025	0.023	−0.208	−0.034	0.033	−0.027	−0.089
Gravidity	−0.858	0.044	0.115	0.096	0.027	−0.047	0.285	0.068
Parity	−0.821	0.117	0.096	0.063	−0.031	−0.002	0.296	−0.048
Miscarriage	−0.557	−0.140	0.067	0.068	0.048	−0.107	0.092	0.095
Medical anamnesis								
History of smoking	0.031	0.100	−0.040	−0.007	−0.582	0.055	−0.082	0.011
History of pelvic surgery	−0.172	0.122	−0.102	−0.021	0.025	0.050	0.827	0.066
Number of previous surgeries	−0.130	0.095	−0.008	0.026	−0.041	0.010	0.904	−0.029
History of salpingectomy and/or oophorectomy	0.038	0.094	−0.014	0.028	0.038	0.353	0.256	0.104
History of appendectomy	0.052	−0.023	0.012	0.101	−0.127	−0.059	0.457	0.128
History of cystectomy	0.063	0.028	−0.419	0.058	0.036	−0.063	0.204	0.125
History of ovarian and/or tubal detorsion	0.024	0.047	0.089	0.034	0.071	0.066	0.131	−0.028
History of laparoscopic tubal ligation	−0.072	0.098	−0.035	0.037	0.032	−0.024	0.056	−0.062
History of other surgeries	−0.340	−0.010	0.059	−0.014	0.031	0.029	0.023	−0.004
Number of previous cesarean sections	−0.131	0.074	0.064	−0.034	−0.048	−0.032	0.781	−0.110
History of cesarean section	−0.194	0.089	0.042	−0.121	−0.006	−0.010	0.802	−0.046
History of endometriosis	0.049	0.109	−0.096	0.001	0.022	−0.025	−0.073	0.027
History of OCP	0.121	0.091	−0.476	0.040	0.037	−0.145	−0.013	0.081
History of PID	0.107	−0.018	0.051	0.080	−0.583	0.222	0.025	0.202
History of ovarian torsion	0.080	0.057	0.055	0.038	0.072	0.186	0.161	−0.069
Comorbidities								
Number of comorbidities	−0.163	0.176	−0.298	−0.009	0.130	0.037	0.373	0.703
Normal uterine pregnancy	−0.018	0.015	0.132	0.146	0.159	−0.040	0.146	0.537
Sleeve gastrectomy	−0.108	−0.068	−0.040	−0.259	−0.010	−0.033	−0.153	0.399
PCOS (includes all cases of “history of infertility”)	0.109	0.096	−0.391	−0.037	−0.118	−0.131	−0.104	0.014
IVF	0.087	0.059	0.012	−0.082	−0.135	0.058	−0.078	0.337
IVF with leading OHSS	−0.001	0.127	−0.027	0.086	0.133	0.098	0.142	0.272
Pelvic mass (ovarian cyst and other masses)	−0.210	0.058	−0.207	−0.047	0.187	0.113	−0.018	0.413
IUCD	−0.145	0.029	0.042	0.085	0.005	−0.118	−0.109	0.094
Endometriosis	0.114	0.048	−0.082	0.043	0.024	−0.008	0.000	0.214
Obesity	0.062	0.054	−0.507	−0.009	0.016	0.037	0.005	0.098
Metabolic syndrome	−0.313	0.116	0.001	0.039	0.002	0.033	−0.124	−0.096
History of pelvic surgery	−0.158	0.122	−0.069	−0.032	0.027	0.069	0.850	0.090
Major symptoms								
Number of major symptoms	−0.008	0.094	0.040	−0.760	0.044	0.011	0.048	−0.083
Flank pain	−0.064	−0.001	0.091	−0.043	0.038	−0.058	−0.036	0.095
Abdominal distension	0.080	0.137	0.047	−0.791	0.009	−0.005	0.028	−0.050
Epigastric pain	0.134	0.054	0.082	0.096	−0.225	0.025	0.121	0.316
Generalized abdominal pain	0.110	−0.124	0.053	−0.298	−0.046	−0.187	−0.039	0.143
Unilateral lower abdominal pain	−0.158	0.145	−0.192	0.164	0.244	0.358	0.059	−0.400
Pelvic pain	0.070	−0.175	0.133	−0.007	−0.230	−0.326	−0.052	0.260
Associated symptoms								
Number of associated symptoms	0.049	−0.935	−0.080	0.030	−0.052	0.130	−0.105	−0.093
Nausea	0.028	−0.717	−0.020	0.106	0.063	−0.123	0.007	−0.053
Vomiting	0.056	−0.786	0.143	0.011	0.086	−0.013	−0.198	−0.033
Fainting	−0.229	−0.172	−0.072	−0.337	0.018	−0.001	−0.109	0.208
Vaginal discharge	−0.127	0.013	−0.017	0.002	−0.852	−0.090	0.150	−0.123
Constipation	0.070	−0.153	−0.178	0.088	0.081	0.038	0.043	−0.198
Diarrhea	0.032	−0.003	0.027	−0.167	−0.024	−0.013	−0.054	0.105
Dysuria	0.000	−0.083	−0.813	0.046	−0.012	0.002	−0.046	−0.116
Fever	0.032	−0.146	0.075	0.053	−0.127	0.818	−0.088	0.053
Vaginal bleeding	−0.127	0.013	−0.017	0.002	−0.852	−0.090	0.150	−0.123
Shivering, shortness of breath	0.039	−0.195	0.093	0.052	−0.102	0.834	−0.076	0.055
Loss of appetite	−0.079	−0.032	0.001	−0.814	0.050	0.035	0.084	0.007
Doppler findings	−0.108	0.108	0.065	−0.180	0.074	0.228	−0.123	0.005
Disease management	−0.270	−0.027	−0.058	−0.223	−0.002	0.245	−0.086	−0.064
Explained variance	4.083	4.140	2.718	2.565	2.634	2.376	4.663	4.083
Total	0.065	0.066	0.043	0.041	0.042	0.038	0.074	0.065

### Results

#### Principal component analysis

Eight principal factors explaining 41.84% of the total variance were extracted (Table 2):

- Factor 1: Demographic and reproductive history (age, parity, gravidity, menopause).
- Factor 2: Acute GI and systemic symptoms (nausea, vomiting, total associated symptoms).
- Factor 3: Genitourinary/metabolic symptoms (dysuria, obesity).
- Factor 4: Acute abdominal symptoms (abdominal distension, loss of appetite).
- Factor 5: Vaginal symptoms (discharge and bleeding).
- Factor 6: Fever and systemic inflammatory features (fever, shivering).
- Factor 7: Surgical history (previous surgeries, cesarean sections).
- Factor 8: Comorbidity and reproductive outcome burden.

These findings underscore the heterogeneity of ovarian torsion presentations and support the multidimensional clinical nature of the disease.

#### Cluster analysis

**Figure 1:**
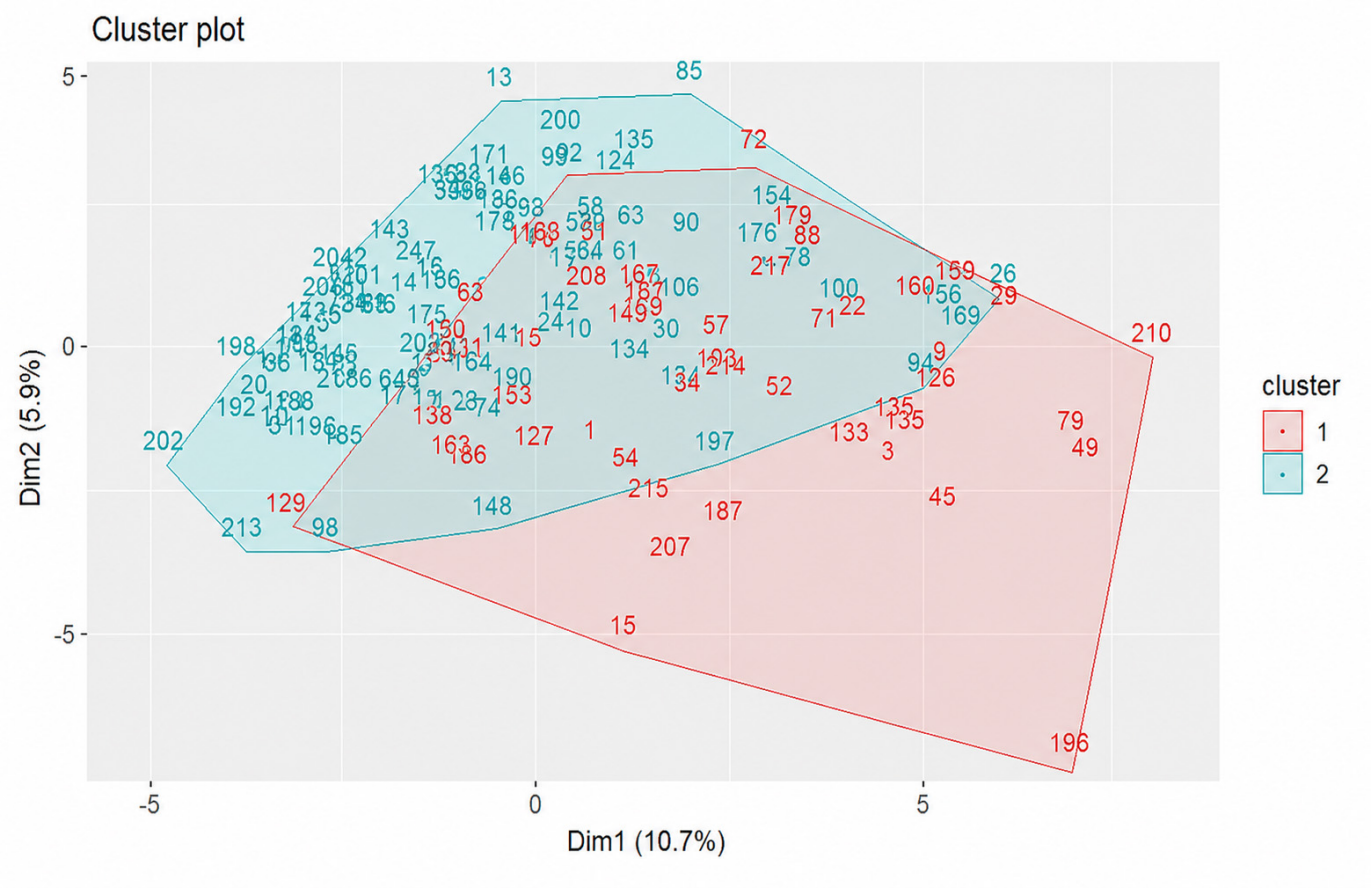
Two-dimensional cluster plot based on principal component analysis (PCA).

Figure 1 shows a cluster plot of ovarian torsion patients based on the first two principal components (Dim1 = 10.7% of explained variance; Dim2 = 5.9%). Each point represents an individual patient, with colors indicating cluster membership (Cluster 1 in red; Cluster 2 in teal). Shaded polygons outline the convex hull of each cluster. Moderate separation is observed primarily along Dim1, with partial overlap between groups, reflecting phenotypically distinct yet clinically overlapping patient profiles.

**Figure 2:**
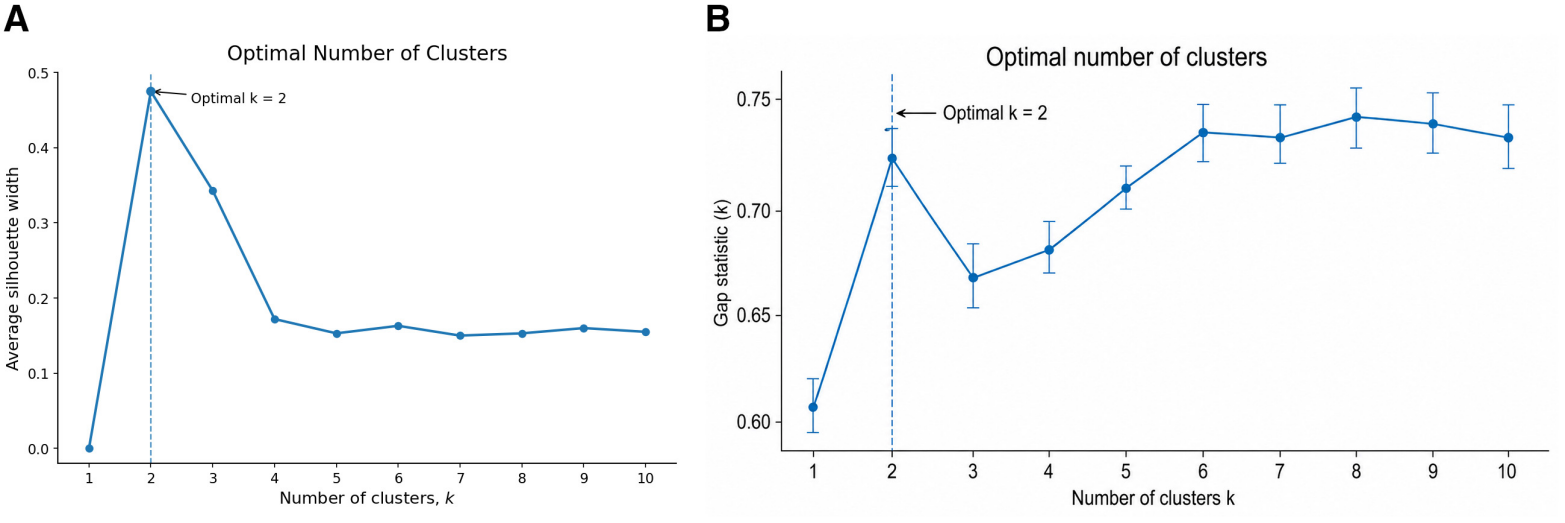
**(A)** Silhouette method for optimal cluster number determination. **(B)** Gap statistic method for optimal cluster number determination.

Figure 2A shows the Silhouette analysis for determining the optimal number of clusters. The average silhouette width is plotted against the number of clusters (k). The highest silhouette value is observed at *k* = 2, indicating that two clusters provide the most appropriate separation and internal cohesion within the dataset. While Figure 2B shows the Gap statistic analysis for optimal cluster selection. The gap statistic values are shown for increasing numbers of clusters (k), with error bars representing standard deviation. The dashed vertical line indicates *k* = 2 as the optimal number of clusters, supporting the silhouette analysis results and confirming the selection of two distinct patient clusters.

Cluster comparison (Table 3) demonstrated statistically significant differences in reproductive history and comorbidity profiles. While age did not differ significantly (*p* = 0.133), gravidity was higher in Cluster 1 (1.94 ± 2.80 vs. 1.08 ± 1.74; *p* = 0.023), with a trend toward higher parity (*p* = 0.078).

**Table 3:** Statistics on different clusters in the study.

Variable	Cluster 1	Cluster 2	*p-value*
Age	28.872 ± 12.387	26.250 ± 12.365	0.133
Gravidity	1.9412 ± 2.7984	1.0833 ± 1.7361	0.023
Parity	1.3529 ± 2.1384	0.8333 ± 1.3889	0.078
No of previous surgeries	0.3529 ± 0.8243	0.3333 ± 0.7244	0.805
No of previous cesarean sections	0.2059 ± 0.6871	0.1875 ± 0.641	0.986
Comorbidities			
No of comorbidities	1.0235 ± 0.9163	0.9375 ± 0.9765	0.448
Endometriosis	0.00%	2.08%	< 0.001
Obesity	0.00%	4.17%	< 0.001
History of sleeve gastrectomy	8.33%	0.00%	< 0.001
History of smoking	0.00%	4.17%	< 0.001
History of salpingectomy and/or oophorectomy	0.00%	4.17%	< 0.001
History of cystectomy	0.00%	4.17%	< 0.001
History of laparoscopic tubal ligation	0.00%	2.08%	< 0.001
History of pelvic inflammatory disease	0.00%	2.08%	< 0.001
Major symptoms			
No of major symptoms	1.0294 ± 0.1695	1.0625 ± 0.2446	0.285
Flank pain	2.08%	0.00%	< 0.001
Fainting	2.08%	0.00%	< 0.001
Constipation or diarrhea	0.00%	6.25%	< 0.001
Associated symptoms			
No of associated symptoms	0.6118 ± 0.6361	0.7917 ± 0.6174	0.061
Fever, shivering, shortness of breath	2.08%	4.17%	< 0.001

The Mann-Whitney U-test was used to compare the distributions of continuous variables between clusters, as the data violated assumptions of normality (assessed via Shapiro-Wilk tests) and homogeneity of variance (assessed via Levene's tests). Pearson's chi-square test of independence was used to evaluate associations between categorical variables and cluster membership.

Although total comorbidity count was similar (*p* = 0.448), Cluster 2 showed significantly higher frequencies of specific conditions, including endometriosis, obesity, smoking history, prior salpingectomy or oophorectomy, cystectomy, laparoscopic tubal ligation, and pelvic inflammatory disease (all *p* < 0.001). In contrast, prior sleeve gastrectomy was more frequent in Cluster 1 (8.33%, *p* < 0.001).

Symptom distribution differed despite similar total major symptom counts (*p* = 0.285). Cluster 1 demonstrated isolated flank pain and fainting (*p* < 0.001), whereas Cluster 2 had higher rates of gastrointestinal symptoms (constipation/diarrhea, *p* < 0.001) and systemic inflammatory features (*p* < 0.001). The number of associated symptoms trended higher in Cluster 2 (*p* = 0.061).

Importantly, management strategy did not differ significantly between clusters.

Overall, the two clusters represent phenotypically distinct yet partially overlapping clinical profiles within the ovarian torsion cohort. A higher burden of comorbidities, especially endometriosis, obesity, and inflammatory symptoms (fever, shivering, SOB) was observed in this cluster. Also notable was a higher incidence of constipation and diarrhea, as well as a slightly higher number of associated symptoms (*p* = 0.061).

### Clinical interpretation of factor structure

Principal Component Analysis (PCA) with Varimax rotation extracted eight factors with eigenvalues greater than ≥1.0, collectively explaining 41.84% of the total variance in the dataset. The first factor (Factor 1) had the highest eigenvalue (6.76) and accounted for 10.73% of the variance alone, while the remaining factors contributed between 3.6% and 5.9% each.

- Factor 1, representing age-related reproductive and demographic characteristics, showed strong negative loadings for age (−0.796), menopause (−0.686), gravidity (−0.858), and parity (−0.821), indicating that these variables cluster strongly and may reflect a reproductive aging dimension.
- Factor 2 was heavily influenced by acute gastrointestinal and systemic symptoms, with strong negative loadings for nausea (−0.717), vomiting (−0.786), and the total number of associated symptoms (−0.935).
- Factor 3 loaded significantly on variables such as dysuria (−0.813) and obesity (−0.507), suggesting a genitourinary or metabolic symptom cluster.
- Factor 4 was defined by acute abdominal symptomatology, with strong negative associations with abdominal distension (−0.791), number of major symptoms (−0.760), and loss of appetite (−0.814), possibly representing an acute inflammatory or pain-related factor.
- Factor 5 loaded strongly on vaginal discharge and bleeding (−0.852), indicating a distinct gynecologic symptom component.
- Factor 6 captured fever-related systemic symptoms, with fever (0.818) and shivering/shortness of breath (0.834) as dominant indicators.
- Factor 7 encompassed prior pelvic surgical history, loading highly on the number of previous surgeries (0.904), history of pelvic surgery (0.827), and cesarean sections (0.781–0.802).
- Factor 8 loaded most prominently on comorbidity burden, including number of comorbidities (0.703) and history of normal pregnancy (0.537), suggesting a general health and reproductive outcome dimension.

### Objective 4: build a model to prognosticate the disease management plan (operative vs. conservative)

#### Methodology

Working on the fourth task, we trained machine learning models to predict the disease management plan. The model architecture we used is as follows: Random Forest, Decision Tree, Logistic Regression, CatBoost, and Neural Network. The major challenge was that the operative cases substantially outnumbered conservative cases.

**Figure 3:**
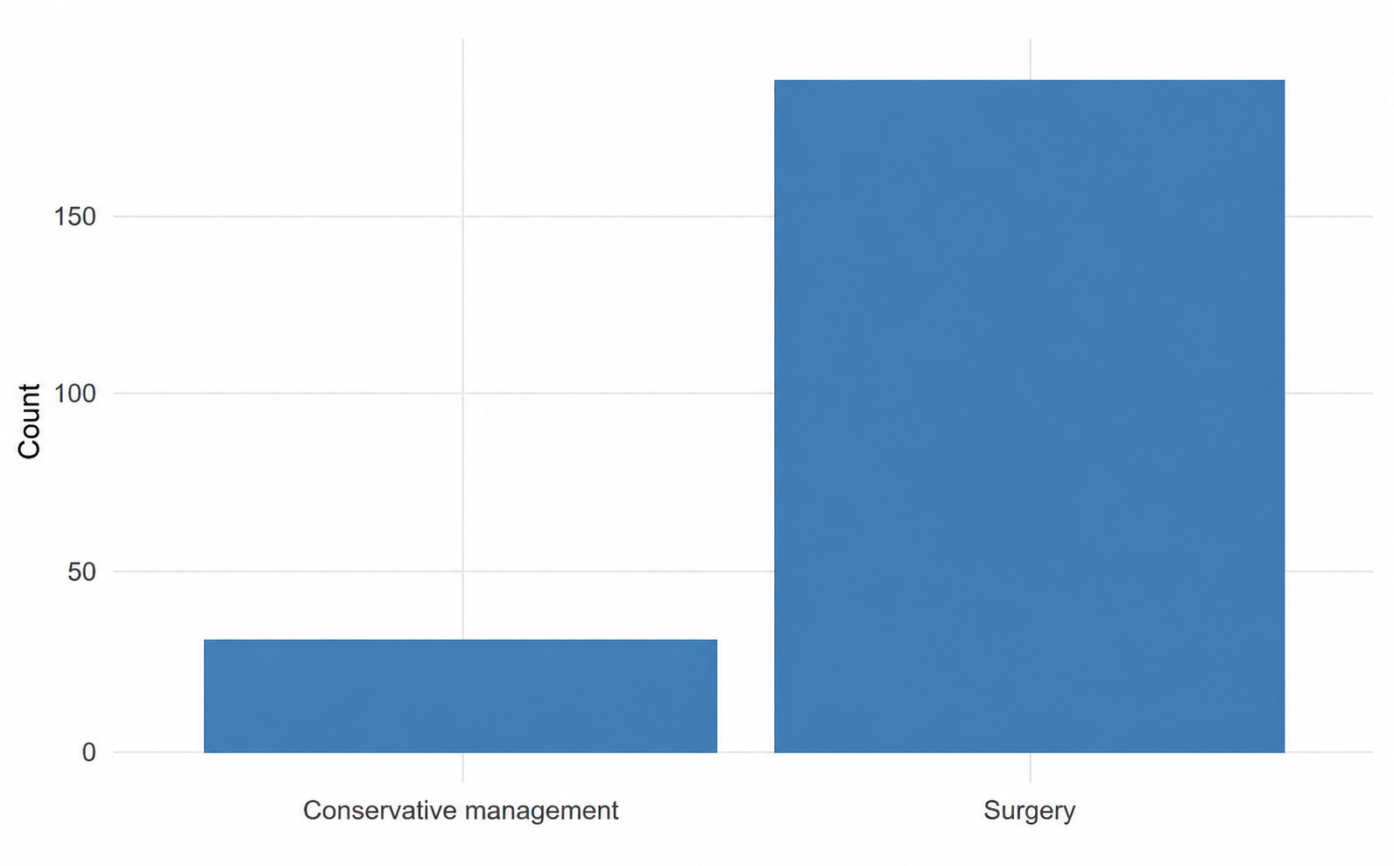
Visualization of class imbalance in the study dataset for treatment modalities.

**Figure 4:**
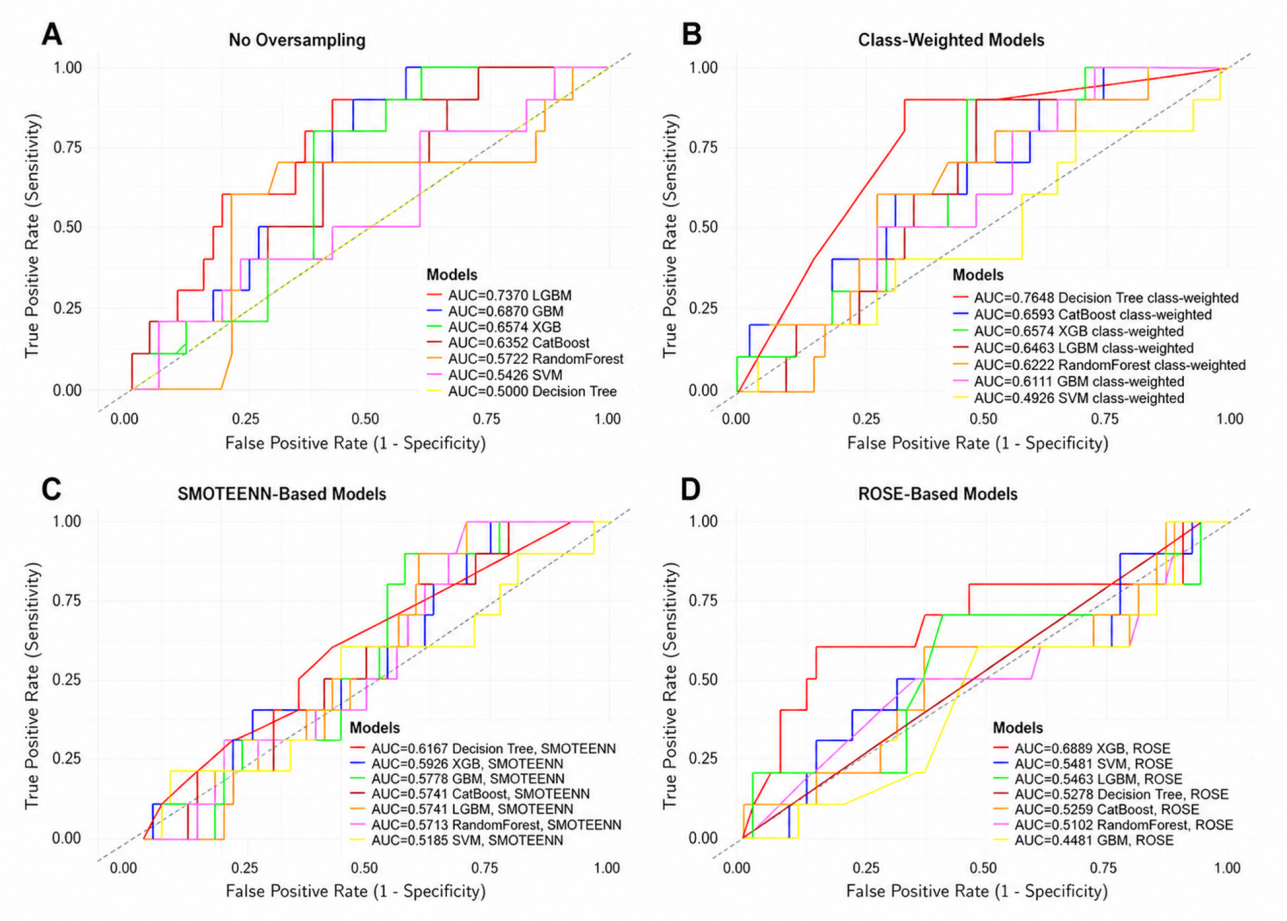
Receiver operating characteristic (ROC) curves comparing machine learning models under different sampling strategies: **(A)** baseline models, **(B)** class-weighted models, **(C)** SMOTE-ENN models, and **(D)** ROSE models.

**Figure 5:**
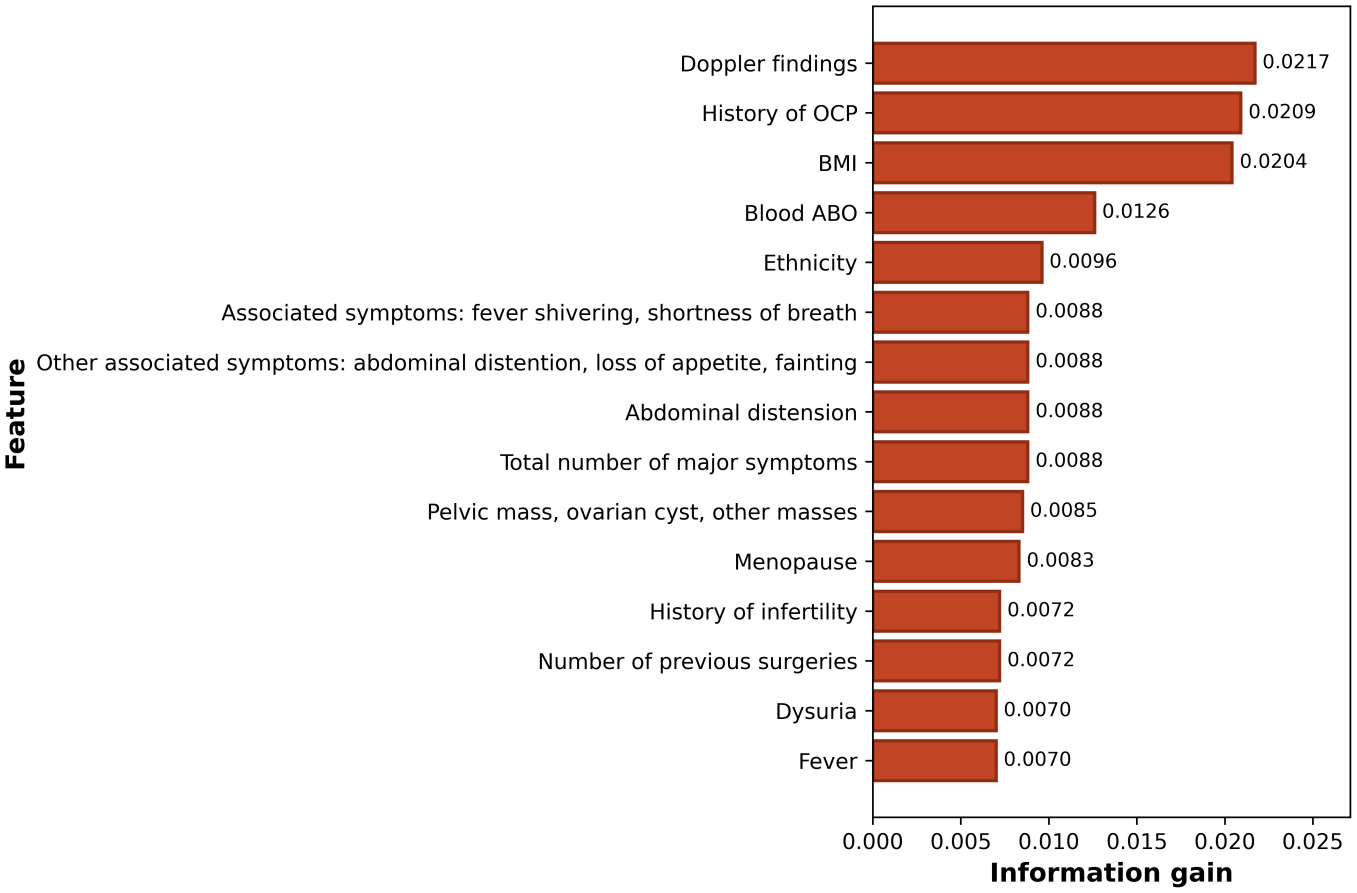
Top 15 predictive features ranked by information gain for Decision Tree model (class weighting).

To address the imbalance in the data, we used data balancing techniques (see Figure 3). These were class weighting, Random Over-Sampling Examples (ROSE), Synthetic Minority Over-sampling Technique and Edited Nearest Neighbors (SMOTE-ENN). The performance metrics were the area under the receiver operating characteristic curve (ROC AUC), sensitivity, and specificity of detecting the positive class - patients with conservative treatment (see Figure 4). To assess feature importance, we computed information gain and ranked predictors by importance (see Figure 5). Table 4 compares the sensitivity and specificity of these models, and Table 5 provides a technique-level summary on imbalance-handling strategies.

**Table 4:** Comparison of model-level AUC with different imbalance-handling strategies.

Model	No oversampling	Class weighting	ROSE	SMOTEENN	Best technique	Best AUC	Gain vs no oversampling
Decision Tree	0.5000	0.7648	0.5278	0.6167	Class weighting	0.7648	0.2648
LGBM	0.7370	0.6463	0.5463	0.5741	No oversampling	0.7370	0.0000
XGB	0.6574	0.6574	0.6889	0.5926	ROSE	0.6889	0.0315
GBM	0.6870	0.6111	0.4481	0.5778	No oversampling	0.6870	0.0000
CatBoost	0.6352	0.6593	0.5259	0.5741	Class weighting	0.6593	0.0241
Random Forest	0.5722	0.6222	0.5102	0.5713	Class weighting	0.6222	0.0500
SVM	0.5426	0.4926	0.5481	0.5185	ROSE	0.5481	0.0055

**Table 5:** Technique-level summary on imbalance-handling strategies.

Technique	Mean AUC	Best single-model AUC
Class weighting	0.6362	0.7648
No oversampling	0.6188	0.7370
SMOTEENN	0.5750	0.6167
ROSE	0.5422	0.6889

#### Results

From our data, no single strategy was best for every model. Class weighting gave the strongest overall average result. It worked especially well for the Decision Tree and Random Forest. ROSE helped XGB and slightly helped SVM. It did not help GBM, LGBM, CatBoost, or Random Forest in this run. SMOTE-ENN improved the Decision Tree relative to no oversampling (0.6167 vs 0.5000), but it did not exceed the best class-weighted result (0.7648). The best model was the class-weighted Decision Tree. This likely happened because the weighted split criterion shifted the tree toward the minority class without adding synthetic noise.- LGBM and GBM were already strong without oversampling. That suggests these boosted models captured the available structure well from the original data.

Model performance varied substantially (Figure 4, Table 4). Decision Tree (Weighted) was the best balanced model (AUC = 0.76), and class weighing was the best technique for handling the imbalanced dataset (Table 5). Top predictors by information gain (Table 6) included doppler findings, BMI, blood group (ABO), ethnicity, major and associated clinical symptoms, the presence of pelvic mass, menopause, history of OCP, infertility and previous surgery. Higher information gain means the feature reduces uncertainty about the treatment outcome more strongly. These features should be viewed as strong screening signals for the class-weighted Decision Tree, not as proof of causal effect. The ranking supports which variables deserve closer clinical review and priority in simplified triage models.

**Table 6:** Top Predictors by Information Gain (Decision Tree + class weighting).

Rank	Feature	Information gain	Unique bins
1	Doppler findings	0.0217	3
2	History of OCP	0.0209	2
3	BMI	0.0204	5
4	Blood ABO	0.0126	4
5	Ethnicity	0.0096	3
6	Associated symptoms: fever shivering, shortness of breath	0.0088	2
7	Other associated symptoms: abdominal distention, loss of appetite, fainting	0.0088	2
8	Abdominal distension	0.0088	2
9	Total number of major symptoms	0.0088	2
10	Pelvic mass, ovarian cyst, other masses	0.0085	2
11	Menopause	0.0083	2
12	History of infertility	0.0072	2
13	Number of previous surgeries	0.0072	2
14	Dysuria	0.0070	2
15	Fever	0.0070	2

## Discussion

Ovarian torsion remains a complex gynecologic emergency in which management decisions must often be made rapidly despite heterogeneous clinical presentations and imperfect diagnostic certainty (13). While prompt surgical intervention is essential in many cases to preserve ovarian viability, unnecessary operative management may expose patients—particularly those of reproductive age—to avoidable surgical risks and potential impacts on fertility (3, 14). In this context, tools that support individualized, evidence-informed decision-making may help optimize the balance between timely intervention and fertility-preserving care.

### Machine learning performance and overfitting considerations

In the present study, multiple machine learning models were evaluated to distinguish between operative and conservative management in ovarian torsion. Besides, we applied different imbalance-handling strategies and collected data on the performance of models trained with class weighting, ROSE, and SMOTE-ENN across the same classification models created without oversampling. Each strategy for treating class-imbalance we tested has certain advantages and disadvantages. The conservative-management group represented a small minority of the cohort, and the combination of class imbalance, high-dimensional predictor space, and synthetic oversampling likely enabled the ensemble model to memorize training patterns rather than learn generalizable relationships. This phenomenon has been well described in small, imbalanced clinical datasets, where overly flexible models may yield unrealistically optimistic performance metrics that fail to replicate in external populations (6, 8).

The class-weighted Decision Tree model demonstrated a more modest yet plausible discriminative ability (AUC = 0.76, sensitivity = 0.75, specificity = 0.73). Importantly, this model did not rely on synthetic data augmentation and provided transparent, human-interpretable decision pathways. In high-stakes emergency scenarios such as ovarian torsion, this balance between performance, robustness, and interpretability is critical, as clinicians must be able to understand and trust the reasoning underlying model outputs (7). Accordingly, the Decision Tree was selected as the most clinically appropriate candidate for future validation. Class weighting keeps all original observations. It is simple to implement and often gives stable gains. This is important because ROSE and SMOTE-ENN showed lower average performance across models, suggesting less stable generalization. We therefore favored the class-weighted Decision Tree as the best-balanced model, because it combined the highest discrimination with a lower overfitting risk and better clinical interpretability.

### Clinical interpretation of predictive features

Analysis of feature importance and correlations revealed several clinically intuitive predictors associated with management decisions. Conservative management was more frequently observed among patients with a history of oral contraceptive pill use, fewer associated symptoms, and absence of a pelvic mass. Conversely, operative management was associated with higher symptom burden and features such as epigastric pain, fever, dysuria, smoking history, and prior adnexal surgery, including salpingectomy or oophorectomy. These findings are consistent with prior literature suggesting that systemic inflammatory features, complex symptomatology, and structural adnexal abnormalities increase concern for compromised ovarian viability and lower the threshold for operative intervention (2, 9).

Notably, Doppler flow findings alone were not determinative of management strategy. This observation aligns with established evidence that preserved Doppler flow does not exclude clinically significant ovarian torsion, particularly in cases of intermittent or partial torsion (10, 11). Management decisions therefore reflect an integrated clinical assessment rather than reliance on any single diagnostic variable.

### Phenotypic heterogeneity revealed by PCA and cluster analysis

Principal component analysis identified eight latent clinical domains underlying ovarian torsion presentations, capturing dimensions such as reproductive aging, acute gastrointestinal and abdominal symptoms, systemic inflammatory response, genitourinary and metabolic features, comorbidity burden, and prior pelvic surgery. These domains reflect the multifactorial nature of ovarian torsion pathophysiology, in which adnexal mobility, hormonal milieu, chronic pelvic pathology, and systemic response interact to shape clinical presentation (12).

Silhouette and gap statistic analyses (Figures 2A, B) consistently identified two optimal clusters, indicating that ovarian torsion presentations in this cohort are not homogeneous but instead group into two distinct phenotypic profiles. Clinically, this finding supports the concept that ovarian torsion encompasses diverse constellations of demographic characteristics, comorbidities, and symptom patterns rather than represents a single uniform entity.

The two-cluster solution suggests one subgroup characterized by lower comorbidity burden and fewer systemic inflammatory features, and a second subgroup enriched for metabolic and inflammatory risk factors, including obesity, endometriosis, prior adnexal surgery, and symptoms such as fever and shivering. Although cluster membership was not independently associated with operative vs. conservative management, the reproducibility of this pattern highlights meaningful clinical heterogeneity within ovarian torsion cases.

Detailed characteristics presented in Table 3 further clarify these distinctions. While age distribution was similar between clusters, Cluster 1 demonstrated higher gravidity and a trend toward higher parity, suggesting greater cumulative reproductive exposure. In contrast, Cluster 2 showed a concentration of metabolic and inflammatory conditions, including obesity, smoking history, and prior adnexal surgery.

These factors are biologically plausible contributors to altered adnexal mobility, pelvic adhesions, and inflammatory milieu—mechanisms that may influence torsion susceptibility and symptom expression. The higher prevalence of inflammatory and gastrointestinal symptoms within Cluster 2 further suggests a more systemically reactive phenotype.

Importantly, although overall comorbidity counts were comparable, the specific types of comorbidities differed substantially, underscoring that disease heterogeneity may be driven by pathophysiological patterns rather than numeric comorbidity burden alone. The absence of significant differences in management strategy between clusters indicates that these phenotypes reflect clinical presentation rather than directly determining treatment decisions.

From a modeling standpoint, the identification of reproducible phenotypic subgroups supports the integration of multidimensional feature interactions into machine learning frameworks. Incorporating composite clinical patterns—rather than isolated variables—may enhance risk stratification and improve generalizability in future prospective validation studies.

### Illustrative clinical decision support pathways

**Figure 6:**
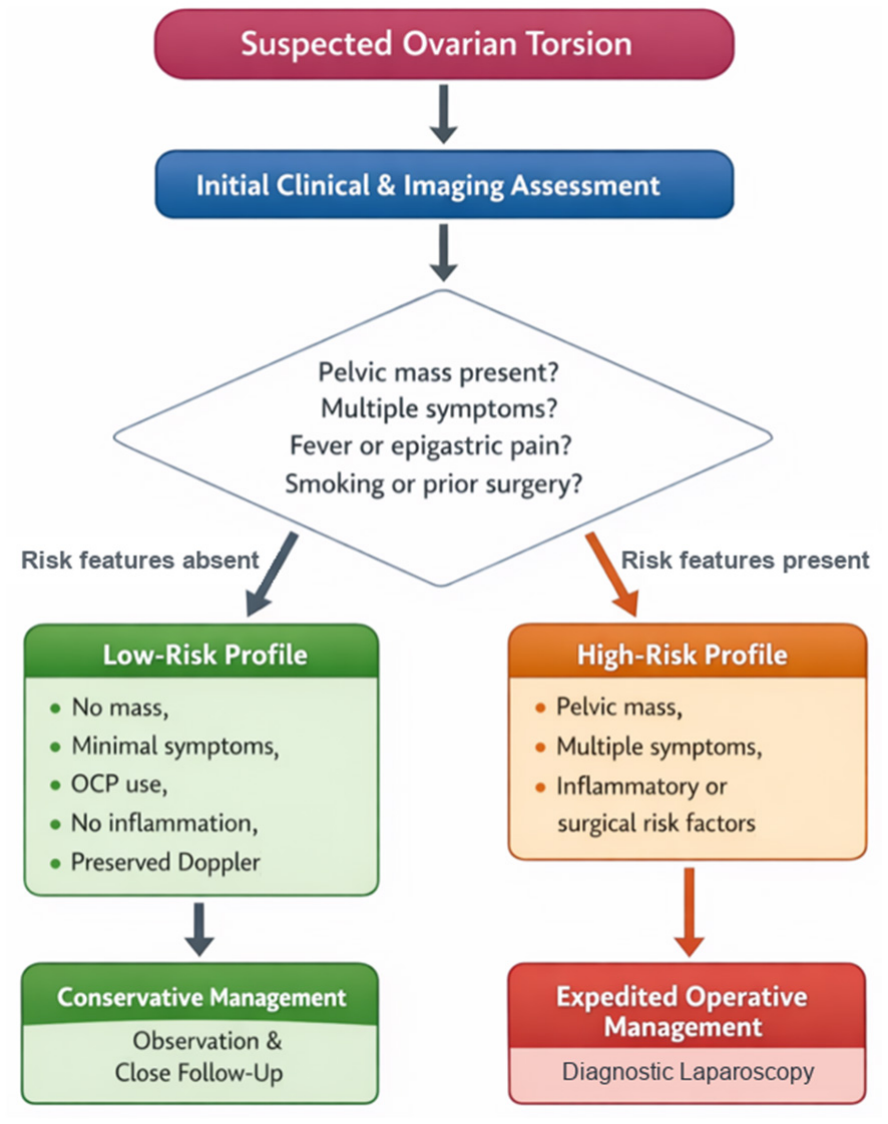
Clinical decision algorithm for suspected ovarian torsion.

While this retrospective study was not designed to establish definitive management thresholds, the consistent patterns observed across correlation analyses, feature importance rankings, and the Decision Tree provide insight into how such a tool might support clinical decision-making. We provide a clinical decision algorithm for suspected ovarian torsion (Figure 6). For example, a patient presenting with suspected ovarian torsion who lacks a pelvic mass, reports minimal associated symptoms, has a history of oral contraceptive use, and demonstrates no systemic inflammatory features may be assigned a relatively low predicted probability of requiring operative intervention. In selected cases, such patients could be considered for short-term observation with close clinical and ultrasonographic follow-up, provided that Doppler flow is preserved and there is no clinical deterioration (2).

Conversely, patients presenting with a pelvic mass, multiple associated symptoms, and one or more high-risk features—such as fever, epigastric pain, smoking history, or prior adnexal surgery—would be assigned a higher predicted probability of requiring surgery and may warrant expedited operative management. These examples are intended to be hypothesis-generating and illustrative rather than prescriptive, and clinical judgment remains essential in all cases.

### Future directions and prospective validation

Before clinical implementation, prospective multicenter validation is essential. A pragmatic cohort study enrolling consecutive patients with suspected ovarian torsion across multiple emergencies and gynecologic centers would allow assessment of model generalizability and calibration. The Decision Tree model derived in this study should be pre-specified and frozen prior to deployment, with standardized collection of predictor variables at presentation. Primary outcomes would include discriminative performance for operative vs. conservative management, with particular emphasis on safely identifying patients who may avoid surgery without compromising ovarian salvage (3).

Secondary outcomes should include ovarian preservation rates, time to surgery, complication rates, frequency of negative or diagnostic laparoscopy, recurrence, and fertility-related outcomes. Anticipated challenges include center-level variability in management thresholds and ensuring adequate representation of conservatively managed cases. Addressing these challenges will be essential to determine whether integration of such a model into clinical decision support systems can meaningfully improve outcomes for patients with ovarian torsion.

## Conclusion

This study demonstrates the potential value of machine learning approaches for supporting individualized management decisions in ovarian torsion, a clinical scenario characterized by diagnostic uncertainty and heterogeneous presentation. While highly flexible models combined with synthetic oversampling may achieve near-perfect apparent performance, such results are likely to reflect overfitting in small, imbalanced clinical datasets and are unlikely to generalize to real-world settings.

The class-weighted Decision Tree model provided a balanced combination of discrimination, robustness, and interpretability. By integrating demographic factors, symptom burden, imaging findings, and clinical history, the model reflects the multifactorial nature of ovarian torsion decision-making and aligns with established clinical reasoning. Exploratory PCA and cluster analyses further highlighted distinct clinical domains and phenotypic patterns, emphasizing that ovarian torsion does not represent a uniform entity and that management decisions must account for patient heterogeneity.

Importantly, this model is intended to function as an adjunct to clinical judgment rather than a replacement. Its potential value lies in identifying patients who may safely undergo conservative management and in prioritizing those who require urgent operative intervention, thereby supporting fertility-preserving care when appropriate. Prospective multicenter validation with standardized data collection and clinically meaningful endpoints is essential to confirm generalizability, refine risk thresholds, and determine whether integration into clinical decision support systems can improve outcomes for patients with ovarian torsion.

## Data Availability

The raw data supporting the conclusions of this article will be made available by the authors, without undue reservation.
